# Is Radiomics Growing towards Clinical Practice?

**DOI:** 10.3390/jpm12091373

**Published:** 2022-08-25

**Authors:** Marco Aiello

**Affiliations:** IRCCS SYNLAB SDN, 80143 Naples, Italy; marco.aiello@synlab.it

A recent article [1], which has received considerable attention since its publication, addresses the problem of the reproducibility of radiomics in multicentric studies, critically focusing on the harmonization methods that are present in the literature in particular.

This study is extremely important because it addresses a striking problem related to the clinical translation of radiomics techniques. This issue is crucial for health research, and it is legitimate to ask how much the growing efforts of the scientific community are actually pointing to the adoption of radiomics in clinical practice.

To better understand the development of radiomics, it is important to focus on its growth rate and, above all, which areas of investigation fall under the keyword “radiomics”. The scientific production in radiomics has grown significantly in recent years [2], and significant annual growth of about 178% has been estimated [3]. These numbers testify to the transition of radiomics from an emerging topic to a real river in flood, with research findings having a revolutionary impact on the medical imaging community.

To investigate this phenomenon, however, it is necessary to understand what is meant by “radiomics”. The basic concept originated from so-called radiogenomics about twenty years ago, with the first studies being on the prediction of the sensitivity of radiotherapy based on gene expression [4,5,6,7]. These new investigations have opened doors to the exploration of the space-time heterogeneity of tumors, both at the microscopic and macroscopic levels [8]. Subsequently, ten years later, the concept of “radiomics” was introduced for the first time [9,10], when it was defined as “the high-throughput extraction of large amounts of image features from radiographic images” [10]. On the other hand, in recent years, we have witnessed a clear expansion of this concept, and the term “radiomics” is increasingly being generalized to any type of computational processing of radiological images [11], including machine learning approaches [12], where the radiomic features may not be explicit but can be considered as emergent properties from the trained model.

This broadening of the concept can cause a risky slowdown in the clinical translation of radiomics as understood in the original sense, on which efforts to develop common initiatives and tools are already focused [13,14,15,16]. Rather, it is time to focus validation efforts by narrowing the field by associating radiomics with specific pathologies and clinical outcomes for which its application can demonstrate an effective clinical impact.

In conclusion, the answer to the question in the title is only partially affirmative. The expansion of the concept of radiomics is certainly favoring the growth of a very active interdisciplinary community that virtuously combines information technology and clinical skills; this aspect, however, should not distract us from the path of “classical radiomics”, which deserves to be specifically directed towards clinical validation.
